# MicroRNAs Expression Patterns Predict Tumor Mutational Burden in Colorectal Cancer

**DOI:** 10.3389/fonc.2020.550986

**Published:** 2021-02-09

**Authors:** Jiahao Huang, Haizhou Liu, Yang Zhao, Tao Luo, Jungang Liu, Junjie Liu, Xiaoyan Pan, Weizhong Tang

**Affiliations:** ^1^ Department of Gastrointestinal Surgery, Affiliated Tumor Hospital, Guangxi Medical University, Nanning, China; ^2^ Department of Colorectal and Anal Surgery, The First Affiliated Hospital, Guangxi Medical University, Nanning, China; ^3^ Guangxi Clinical Research Center for Colorectal Cancer, Nanning, China; ^4^ Department of Research, Affiliated Tumor Hospital, Guangxi Medical University, Nanning, China; ^5^ Department of Radiology, Affiliated Tumor Hospital, Guangxi Medical University, Nanning, China; ^6^ Department of Hepatobiliary Surgery, Affiliated Tumor Hospital, Guangxi Medical University, Nanning, China; ^7^ Department of Ultrasound, Affiliated Tumor Hospital, Guangxi Medical University, Nanning, China; ^8^ Department of Comprehensive Internal Medicine, Affiliated Tumor Hospital, Guangxi Medical University, Nanning, China

**Keywords:** tumor mutational burden, microRNA, colorectal cancer, immunotherapy, microsatellite instability

## Abstract

**Background:**

Tumor mutational burden (TMB) could be a measure of response to immune checkpoint inhibitors therapy for patients with colorectal cancer (CRC). MicroRNAs (miRNAs) participate in anticancer immune responses. In the present study, we determined miRNA expression patterns in patients with CRC and built a signature that predicts TMB.

**Methods:**

Next generation sequencing (NGS) on formalin-fixed paraffin-embedded samples from CRC patients was performed to measure TMB levels. We used datasets from The Cancer Genome Atlas to compare miRNA expression patterns in samples with high and low TMB from patients with CRC. We created an miRNA-based signature index using the selection operator (LASSO) and least absolute shrinkage method from the training set. We used an independent test set as internal validation. We used real-time polymerase chain reaction (RT-PCR) to validate the miRNA-based signature classifier.

**Results:**

Twenty-seven samples from CRC patients underwent NGS to determine the TMB level. We identified four miRNA candidates in the training set for predicting TMB (N = 311). We used the test set (N = 204) for internal validation. The four-miRNA-based signature classifier was an accurate predictor of TMB, with accuracy 0.963 in the training set. In the test set, it was 0.902; and it was 0.946 in the total set. The classifier was superior to microsatellite instability (MSI) for predicting TMB in TCGA dataset. In the validation cohort, MSI status more positively correlated with TMB levels than did the classifier. Validation from RT-qPCR showed good target discrimination of the classifier for TMB prediction.

**Conclusion:**

To our knowledge, this is the first miRNA-based signature classifier validated using high quality clinical data to accurately predict TMB level in patients with CRC.

## Highlights

TMB is a biomarker of response to treatment with immune checkpoint inhibitors for patients with CRC. Small tissue amounts obtained using conventional methods limit the use of TMB. Assessment of TMB using miRNA expression patterns may be useful to guide the selection of appropriate immunotherapies.

## Introduction

Colorectal cancer (CRC) is among the most common cancers. In terms of mortality, it is the third-most lethal; 5-year survival rates are approximately 50%; 10-year survival rates are approximately 20% (1–5). Normally, CRC is asymptomatic until it reaches an advanced stage. Unfortunately, as many as 25% present with metastatic disease (6). These findings suggest that more effective treatments are urgently required.

Within the most recent decade, immunotherapy has proven to be remarkably successful in achieving durable responses in solid tumors such as melanoma and lung cancer. Increasing attention has been paid to immune checkpoint inhibitors (ICIs) therapy. ICIs include programmed cell death 1 ligand 1 (PD-L1), cytotoxic T lymphocyte antigen 4 (CTLA-4), and programmed cell death 1 (PD-1). A small subset of patients with metastatic CRC who are mismatch-repair-deficient (dMMR) or who harbor high levels of microsatellite instability (MSI-H) are amenable to such therapy.

Tumor mutation burden (TMB) represents genomic instability; it marks responses to immunotherapy in a variety of tumor types (7, 8). Highly mutated genes harbor the potential to produce neoantigens; in so doing, they improve immunogenicity responses to immunotherapy (9). Importantly, approximately 15% of CRCs and 5% of mCRCs are MSI-H, and are characterized by elevated TMB (10, 11). TMB was initially assessed using next-generation sequencing; nevertheless, tissue obtained *via* surgery and high cost limits the use of tissue-based approaches. These findings suggest that novel predictive biomarkers to distinguish CRCs that respond to immunotherapy are needed.

MicroRNAs (miRNAs) are small, endogenous, 21–23-nucleotide molecules. They take part in post-transcriptional modifications (12, 13). MiRNAs are critical regulators of anticancer immune responses (14, 15). These molecules negatively regulate tumor cell PD-L1 expression, that correlate with successful treatment of CRC (16, 17). Therefore, we hypothesized that the miRNA-TMB axis might predict responses to ICIs in patients with CRC.

In the present study, 27 samples from CRC patients underwent targeted next-generation sequencing (Cancer Sequencing YS panel) to evaluate the TMB and MSI level. We downloaded gene datasets and screened mutation annotation files for differentially expressed miRNAs in CRC from TCGA databases. We determined the value of miRNA-based signature classifiers in prediction of TMB in paraffin-embedded CRC samples using quantitative real-time polymerase chain reaction (qRT-PCR).

## Methods

### Patients and Samples

We analyzed 27 paraffin-embedded, formalin-fixed samples of tissue from CRC patients who had been referred to OrigiMed (Shanghai, China) to undergo targeted next generation sequencing (NGS) test from April 2018 to August 2019. The study was approved by the institutional ethics review committee of Affiliated Tumor Hospital of Guangxi Medical University and First Affiliated Hospital of Guangxi Medical University. Prior to sample collection, each patient provided written informed consent.

### Targeted Next-Generation Sequencing

Genomic DNA from formalin fixed paraffin embedded (FFPE) tumor samples were isolated using the QIAamp DNA FFPE Tissue Kit as instructed by the manufacturer. All coding exons from 578 cancer-related genes and the selected introns of 47 common genes rearranged in solid tumors, which were included in the Cancer Sequencing YS panel (CSYS) were captured and sequenced with a mean coverage of 900X for FFPE samples using the Illumina NextSeq-500 Platform (Illumina Incorporated, San Diego, CA) at OrigiMed (Shanghai, China) (18). The raw sequencing data underwent stringent quality control by examining sequencing coverage and uniformity. A suite of customized bioinformatics pipelines was applied for discovery of short and long indels copy number variations as well as genomic alterations such as single nucleotide variations, in addition to TMB and MSI.

### Tumor Mutational Burden and Microsatellite Instability Analysis

As previously prescribed, TMB score was calculated from CSYS data for each sample by counting the number of somatic mutations, including coding single nucleotide variants (SNVs) and indels, per megabase (Mb) of the sequence examined. Driver mutations and germline alternations from the National Center for Biotechnology Information’s Single Nucleotide Polymorphism Database (dbSNP) were not counted. We applied 1.25 Mb as the coding region size of the CSYS panel. We also calculated the relationship between TMB levels and the clinicopathological features of patients with CRC. There were 572 microsatellite loci (MSLs) identified in the CSYS‐targeted region as candidate MSI markers (18).

### The Cancer Genome Atlas Data Processing

The Cancer Genome Atlas (TCGA) was a landmark cancer genomics program, which molecularly characterized over 20,000 primary cancer and matched normal samples. TCGA as well as mutation annotation files were downloaded from the Genomic Data Commons Data Portal using Data Transfer Tool (https://gdc.cancer.gov/access-data/gdc-data-transfer-tool). We downloaded clinical data and the RNA sequencing expression profiles from the UCSC Xena (https://xenabrowser.net/). We used data based on the cohorts of Colon Cancer (COAD) and Rectal Cancer (READ) in the TCGA. The VarScan pipeline was applied for the somatic mutation calling workflow. We used Maftools to read the somatic variants in each sample (19). We defined TMB as the count of somatic variants per megabase (MB) (20). We defined high TMB level as ≥10 mutations per MB; low TMB level was defined as <10 mutations per MB. We used 38 MB as the estimate of the exome size (21).

**Figure 1:**
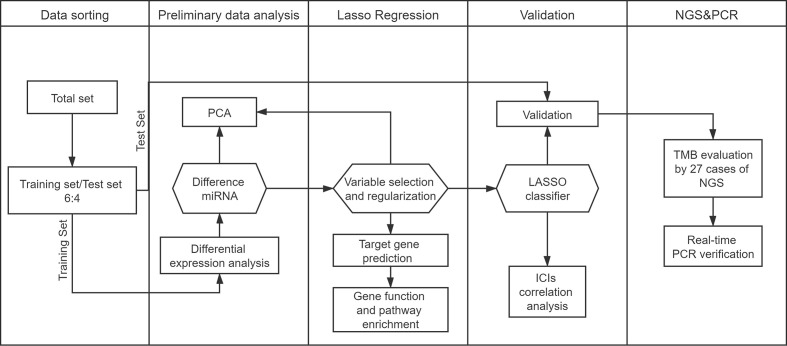
Study workflow.

COAD and READ miRNA mature strand expressions were downloaded from the Genomic Data Commons Data Portal using Data Transfer Tool. Our miRNA expression profiles included 616 samples based on the IlluminaHiSeq_miRNASeq platform (Illumina Inc., San Diego, CA, USA). A total of 515 samples with both miRNA expression profiles and mutation annotation files (388 colon cancer tissue samples and 127 rectal cancer tissue samples) were included in the sample. We randomly assigned samples to the training set (60%) and test set (40%). We illustrate the workflow in **Figure 1**.

### Screen of Differentially Expressed MicroRNAs and Bi-Directional Hierarchical Clustering

We removed all miRNAs from the dataset that were not expressed in >10% of CRC samples. We analyzed the miRNAs that were differentially expressed between high TMB and low TMB samples using the “edgeR” package in R. Fold-changes (FCs) in expression levels of individual miRNAs were also measured. Differentially expressed miRNAs with |log2FC|> 1 and P (adjusted by false discovery rate) values < 0.01 were considered significant. We performed bidirectional hierarchical clustering for the differentially expressed miRNAs based on Euclidean distance. The results were expressed on a heat map.

### Least Absolute Shrinkage and Selection Operator Method and Principal Component Analysis for Feature Selection

We determined expression values of differentially expressed miRNAs for each CRC sample in the training set. The least absolute shrinkage and selection operator (LASSO) method was used to generate strong predictive values and low correlations between each, so as to prevent over-fitting and to select the best features for the high-dimensional data. We then performed LASSO logistic regression model analysis using the “glmnet” package in R. The LASSO method was used to select optimal biomarkers for predicting TMB. Principal component analysis (PCA) was performed prior to feature selection using expression profiles from all differentially expressed miRNAs. We subsequently performed PCA using expression profiles from the optimally differentially expressed miRNAs. Samples in two-dimensional graphs were plotted across the first two principal components.

### Predicting Tumor Mutational Burden Level Using the MicroRNA-Based Signature Classifier

Using the LASSO method, we selected the miRNAs that had been determined to have non-zero regression coefficients. These were considered optimal miRNAs for establishment of the miRNA-based signature classifier for prediction TMB. We then generated a classifier index for each sample using regression coefficients from LASSO analysis to weight expression values of the selected miRNAs. We used the following formula: index = ExpmiRNA1*Coef1 + ExpmiRNA2*Coef2 + ExpmiRNA3 *Coef3+ …, where “Coef” is the regression coefficient of miRNA derived from the LASSO logistic regression and “Exp” is the expression values of the miRNAs. The test set was used to validate the classifier’s transferability and robustness. We assessed the efficiency of the classifier using accuracy, sensitivity (Se), specificity (Sp), positive predictive value (PPV), negative predictive value (NPV), and area under the receiver operating characteristic (ROC) curves. We drew ROC curves and compared them using the “plotROC” package in R. Delong’s test was used to compare the area under the curve (AUC) across various models (22).

### Correlations Between the MicroRNA-Based Signature Classifier and the Expression of Three Immune Checkpoints; Functional Enrichment Analysis

The expression values were normalized in log2(x+1) transformed RSEM count. We calculated the correlations between miRNA-based signature classifier index and expression of various immune checkpoints (PD-1, PD-L1, and CTLA-4). We used the R package’s “clusterProfiler” to analyze gene ontology (GO) enrichment and the Kyoto Encyclopedia of Genes and Genomes (KEGG) pathways. GO terms and KEGG pathways with P-values <.05 were considered to be enriched significantly.

### Target Gene Prediction

After obtaining the miRNA target genes using the predictive target module of miRWalk2.0 online software (http://mirwalk.umm.uni-heidelberg.de), the following parameters were considered: 3´UTR positioning, miRNA seeds starting at position 1 and the minimum seed length was set to 7 bp.

### RNA Isolation

With the aim of investigating the miRNA expressions in the CRC tissues and normal tissue, we cut five 8-μm sections from FFPE tissue samples and deparaffinized them using xylene and ethanol. We extracted total RNA (including miRNAs) from FFPE tissue samples using TRIzol (Thermo Fisher Scientific, US), according to the manufacturer’s protocol. We measured concentrations and purities of the isolated using a spectrophotometer (NanoDrop 2000, NanoDrop Technologies Inc., USA). After isolation, we immediately stored samples at –80°C until use.

### Quantitative Real-Time Polymerase Chain Reaction

Mir-X miRNA qRT-PCR SYBR Kits (Takara Bio Inc., Kusatsu, Japan) were used to synthesize cDNA using specific reverse transcription primers (**Supplementary Table S1**). After reverse transcription, we performed real-time PCR reaction using One Step TB Green^®^ PrimeScript™ RT-PCR Kit (Perfect Real Time) (Takara Bio Inc., Kusatsu, Japan) with reaction volume of 20 μl on 7500 Fast Real-Time PCR (Applied Biosystems, USA). The reaction conditions were as per the manufacturer’s instructions, and each sample was performed in triplicate. MiRNA expression levels were calculated using the 2^−△△Ct^ method (23). The cycle threshold (CT) values of miRNA were normalized to the level of internal reference (RNU6, assay ID 001093).

### Statistical Analysis

We used the χ^2^-test categorical data from IBM SPSS Statistics software version 22.0 (IBM, Armonk, NY, USA). We compared levels of expression of miRNAs in the high TMB and low TMB groups with unpaired t-tests using the edgeR package. We determined statistical relationships between TMB levels and clinicopathological features of CRC patients per variable type. We used Spearman correlation coefficients to determine statistical significances of TMB levels and continuous variables. We used the non-parametric Kendall’s tau-b test for binary variables such as MSI. P-values < 0.01 were considered significant.

## Results

### Patient Enrollment

We assayed tumor tissues obtained from 27 patients with pathologically confirmed CRC to locate genetic alterations and calculate TMB. The inclusion criteria were patients with colorectal adenocarcinoma who were confirmed by pathology, with evaluative TMB data. We summarized demographic and clinical characteristics in **Table 1**. MSI accounted for 10–15% of the CRC patients. To determine the impact of MSI status on TMB values, we created a cohort of CRC samples with MSI-H (seven cases) and MSS (20 cases) to compare TMB with MSI statuses.

**Table 1:** Patient characteristics.

	TMB-high	TMB-low	P-value
	N=9	N=18	
Age			0.691
Median (range, year)	55 (33–93)	52 (34–73)	
Sex			0.100
Male	7	8	
Female	2	10	
Tumor location			0.683
Right-sided colon	3	5	
Left-sided colon	4	6	
Rectum	2	7	
Clinical stage			0.440
I	0	2	
II	5	5	
III	3	7	
IV	1	4	
MSI status	MSI-H (7/9, 77.8%); MSS (2/9, 22.2%)	MSI-H (0/18, 0%); MSS (18/18, 100%)	<0.001

### Tumor Mutational Burden Evaluation Using Targeted Next Generation Sequencing

The median TMB for the study population of 27 CRC patients was 26.2 (0.9–92.9) mutations/Mb (Mut/Mb). As expected, we observed a significant difference in TMB counts between the two subtypes. Patients with MSI-H had significantly higher TMB than those with MSS (*P <*0.0001). Consistent with previous results (24), patients with MSS tumors had low TMB between 0.9 and 9.4 Mut/Mbp and greater TMB-low patient rates (74.1%, 20/27 *vs*. 25.9%, 7/27, *P <*0.001), compared to MSI-H cases with TMB between 16.0 and 92.9 Mut/Mbp. All validated samples underwent MMR-immunohistochemical testing as the gold standard.

### Differentially Expressed MicroRNAs and Bidirectional Hierarchical Clustering in The Cancer Genome Atlas

**Figure 2:**
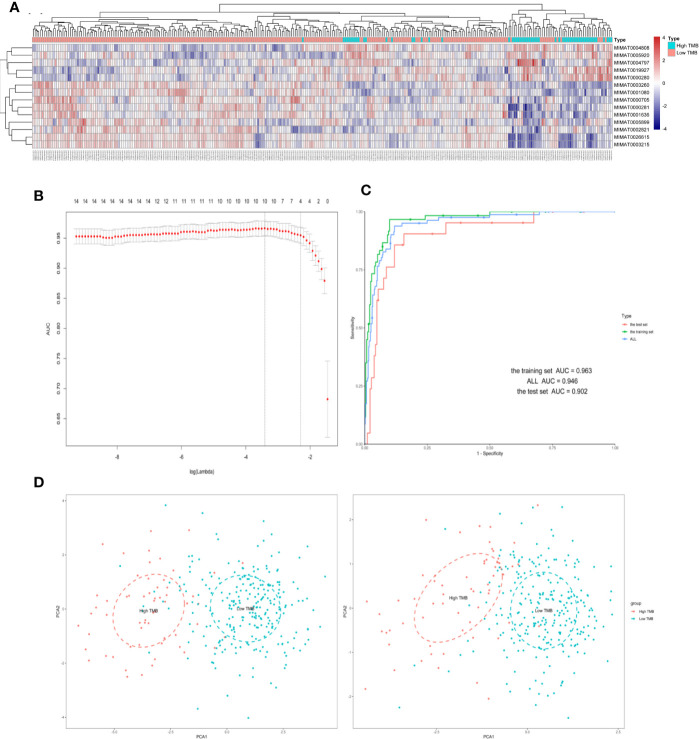
Four differently expressed microRNAs were identified from The Cancer Genome Atlas (TCGA) in samples of colorectal cancer (CRC). **(A)** Hierarchical clustering of expression patterns from differentially expressed microRNAs discriminate between high and low TMB. **(B)** The microRNAs (miRNAs) selection results of the LASSO mode. **(C)** Receiver operating characteristic curve of the training/test set. **(D)** Principal component analysis (PCA) plot of all samples before and after LASSO variable reduction. LASSO, least absolute shrinkage and selection operator.

A total of 515 samples with both miRNA expression profiles and mutation annotation files (388 colon cancer tissue samples and 127 rectal cancer tissue samples) were included in the sample. There were no significant differences in terms of clinicopathological characteristics between the training and test sets (**Table 2**). The training set included 60 samples with high TMB levels and 252 samples with low TMB levels. Fourteen miRNAs were differentially expressed between the high TMB and low TMB level samples using our cut-off criteria (P < 0.01 and |log2FC|> 1). The high TMB group included nine upregulated miRNAs and five downregulated miRNAs. We generated a heat map to display the results of the expression analysis (**Figure 2A**). Hierarchical clustering showed that these differentially expressed miRNAs had expression patterns that distinguished high from low TMB samples.

**Table 2:** Clinicopathological factors of patients with colorectal cancer (CRC) in the the Cancer Genome Atlas (TCGA) dataset.

Characteristics	Training set	Test set	*P* value
	N=311	N=204	
Age (years)			
<60	96 (30.87%)	61 (29.90%)	0.816
≥60	215 (69.13%)	143 (70.10%)	
Gender			
Male	165 (50.05%)	102 (50.00%)	0.498
Female	146 (46.95%)	102 (50.00%)	
Histologic type			
Colon adenocarcinoma	202 (64.95%)	128 (62.74%)	0.054
Colon mucinous adenocarcinoma	41 (13.18%)	14 (6.97%)	
Rectal adenocarcinoma	59 (18.97%)	55 (26.96%)	
Rectal mucinous adenocarcinoma	6 (1.93%)	3 (1.47%)	
Not available	3 (0.96%)	4 (1.96%)	
Lymphatic metastasis			
N0	176 (56.59%)	108 (52.94%)	0.688
N1–2	133 (42.77%)	95 (46.57%)	
NX	2 (0.64%)	1 (0.49%)	
T stage			
T0–2	64 (20.58%)	39 (19.11%)	0.434
T3–4	247 (79.42%)	164 (80.39%)	
Not available	0 (0%)	1 (0.49%)	
M stage			
M0	216 (69.45%)	151 (74.02%)	0.101
M1	44 (14.15%)	33 (16.18%)	
Mx	51 (16.40%)	20 (9.80%)	
Pathologic stage			
I–II	166 (53.38%)	102 (50.00%)	0.288
III–IV	131 (42.12%)	97 (47.55%)	
Not available	14 (4.50%)	5 (2.45%)	
CEA (ng/ml)			
<5.0	122 (39.23%)	82 (40.20%)	0.814
≥5.0	71 (22.83%)	50 (24.51%)	
Not available	118 (37.94%)	72 (35.29%)	

### The Least Absolute Shrinkage and Selection Operator Method for Principal Component Analysis and Feature Selection

We used the LASSO logistic regression method with the expression data from the 14 miRNAs in the training set and generated an miRNA-based signature classifier for TMB in CRC patients. We computed group-wise classifications in 10-fold cross-validations. Type measure = “AUC” was area under the receiver operating characteristic (ROC) curve, which is one of the most popular comprehensive considerations for the performance of the model. We identified four miRNAs with non-zero regression coefficients [**Figure 2B** as optimal features (hsa-miR-592, hsa-miR-625-3p, hsa-miR-552-5p, and hsa-miR-224-5p)]. Lasso regression analysis is a shrinkage and variable selection method. **Figures 2C, D** illustrated the PCA results before and after the processing of LASSO methods, respectively. Finally, we selected four miRNAs from multiple differential miRNAs found using different TMB groupings.

### The Four-MicroRNAs Based Classifier

With LASSO and 10-fold cross-validation, we identified four miRNAs that had non-zero regression coefficients: lambda.min = 0.03299115, and lambda.1se = 0.1105732. We created the miRNA-based classifier index using this formula: index = miR-592*(−0.2271092)+miR-625-3p*(0.2636113)+miR-552-5p*(−0.1849247)+miR-224-5p*(−0.101137). Notably, the four-miRNA-based classifier had a high sample recognition efficiency. For the training, test, and total sets, the specificities were 0.901, 0.881, and 0.881, respectively, and accuracies were 0.963, 0.902, and 0.946, respectively. ROC curve analysis revealed that the AUC was 96.33% (95% CI: 94.09–98.56%) in the training set, and 90.24% (95% CI: 83.33–97.16%) in the test set, without significant difference (Delong method, *P* = 0.102, **Figure 2C**). Results for negative and positive predictive values using the full data set (training set and test set) are shown in the **Supplementary Table S2**.

### Correlation of the Classifier Index with Tumor Mutational Burden and Enrichment Analysis

**Figure 3:**
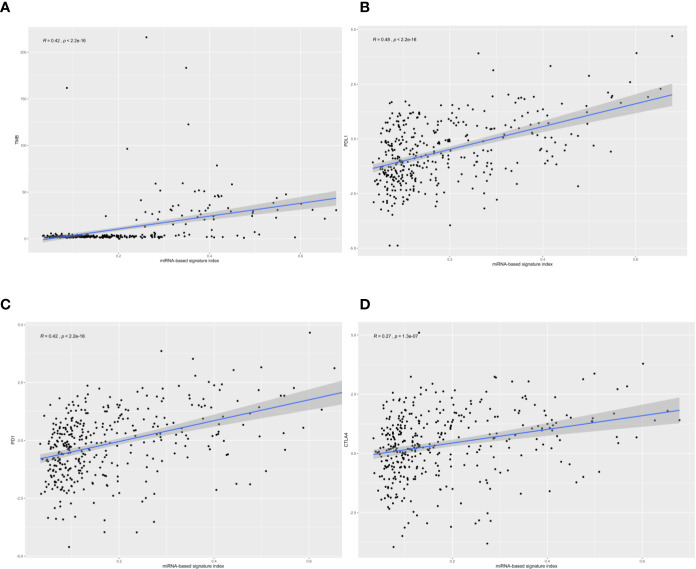
The correlation of four-microRNA (miRNA)-based signature index with tumor mutational burden (TMB), PD-L1, PD-1, and CTLA-4. The four-miRNA-based signature index is **(A)** correlation with TMB; **(B)** low correlation with PD-L1 expression; **(C)** low correlation with PD-1 expression, and **(D)** no correlation with CTLA4 expression.

We calculated the classifier index of all samples in the total set as well as the correlations of the classifier index with TMB and three immune checkpoints. We found that the four-miRNA based classifier index correlated with TMB (Pearson R = 0.42, *P* = 2.2 x 10^−16^, **Figure 3A**), correlated with PD-L1 (Pearson R = 0.48, *P* = 2.2 x 10^−16^, **Figure 3B**) and PD-1 (Pearson R = 0.42, *P* = 2.2 x 10^−16^, **Figure 3C**); there was no correlation with CTLA-4 (Pearson R = 0.27, *P* =1.3 x 10^−7^, **Figure 3D**).

**Figure 4:**
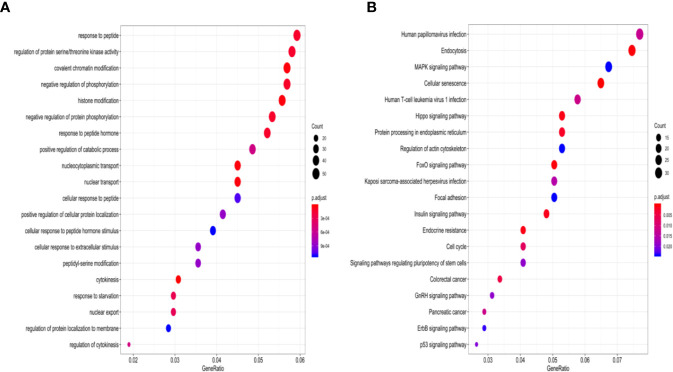
GO enrichment analysis and KEGG pathways enrichment analysis of four-miRNAs targeting mRNAs. **(A)** The top 20 over-represented biological processes are primarily involved. **(B)** Significantly enriched cancer-related KEGG pathways. GO, gene ontology; KEGG, the Kyoto Encyclopedia of Genes and Genomes.

According to StarBase, PD-1, PD-L1, and CTLA-4 were not targeted by the four miRNAs. The TMB levels weakly positively correlated with MSI (correlation coefficient = 0.177, *P <*0.001). The four-miRNA-based classifier tended to moderately positively correlate with TMB levels (Pearson correlation = 0.429, *P <*0.001). Enrichment analysis for these four miRNAs demonstrated that these molecules take part in many immune-related processes (**Figure 4A**) as well as cancer-related pathways (**Figure 4B**).

### MicroRNA Expression Levels in Normal and Colorectal Cancer Tissues

**Figure 5:**
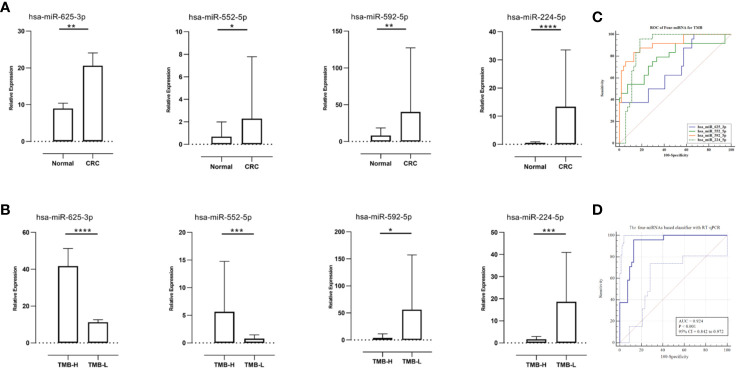
Relative microRNAs (miRNAs) expression in diverse tissues and receiver operating characteristic (ROC) curves for the confirmation of miRNA-based signature index. **(A)** Relative miRNAs expression in paired samples (N = 27). **(B)** The relative miRNAs expression levels were compared between TMB-High CRC samples (N = 9) and TMB-Low CRC samples (N = 18). **(C)** The ROC curves of the single miRNA (qRT-PCR results of 27 samples). **(D)** The ROC curves of the four-miRNA (qRT-PCR results of 27 samples) in the model. *P < 0.05, **P < 0.01, ***P < 0.001 and ****P < 0.0001.

To compare expression levels of miRNAs in TMB-H and TMB-L CRC patients, FFPE tumor samples and tumor-matched non-cancerous tissues were evaluated using RT-qPCR. Expression levels of hsa-miR-625-3p, hsa-miR-552-5p, hsa-miR-592, and hsa-miR-224-5p were significantly different between primary tumors and non-cancerous colorectal tissues. More specifically, we found significant upregulation of the four miRNAs in all sufficiently represented CRC in comparison with non-cancerous controls (*P <*0.01, *P <*0.05, *P <*0.01, and *P <*0.001, respectively, **Figure 5A**).

### Association Between MicroRNA Expression and Tumor Mutational Burden Status

Because the miRNA-TMB axis might predict responses to ICIs in CRC, we determined whether expression of miRNAs and TMB status were related. Expression levels of hsa-miR-625-3p and hsa-miR-552-5p in the patients with TMB-H CRC were significantly higher than TMB-L CRC (*P <*0.0001 and *P <*0.001, respectively, **Figure 5B**). Notably, higher expression levels of hsa-miR-592 and hsa-miR-224-5p were found in TMB-L CRC patients than in TMB-H CRC patients (*P <*0.05 and *P <*0.001, respectively).

Regarding the relationship of TMB levels with clinicopathological features in twenty-seven CRC patients, TMB levels highly positively correlated with MSI status (correlation coefficient = 0.822, *P <*0.001). In addition, the 4 miRNA-based classifier tended to moderate positive correlation with TMB levels (Pearson correlation = 0.625, *P <*0.001).

To verify the robustness of four-miRNA classifier, ROC analysis was performed and AUC was calculated according to results of RT-qPCR. In ROC analysis, the four-miRNA obtained an AUC of 0.924 (accuracy=0.898), which was close to the AUC calculated from TCGA data set (0.946). The single miRNA achieved an AUC (**Figure 5C**) that was lower than the AUC calculated from the total miRNAs (**Figure 5D**). In summary, all four miRNAs provided promising AUC values for predicting TMB levels in patients with CRC.

## Discussion

Immunotherapy has attracted substantial attention based largely on its success in achieving durable responses in several cancer types, including a subset of CRCs (25, 26). Nevertheless, response rates to PD-1 blockade in MSI-H CRC vary and a large proportion of mutant neoantigens in responding tumors are highly sensitive to PD-1 blockade (27). Others have noted significant correlations between TMB and responses to ICIs; these correlations appeared to be independent of MSI status as well as PD-L1 expression (7, 28–30). TMB is determined using NGS methodologies by counting the number of somatic mutations in a sample. The development of additional biomarkers in predicting high TMB which could guide physicians on how to sequence PD-1 inhibitors is an urgent unmet need. Samples of small liquid biopsy may not represent true TMB, especially in the context of intratumoral heterogeneity. Because miRNAs play substantial key roles in CRC tumor immunity (31, 32), we investigated the association of miRNA expression patterns with TMB levels in patients with CRC.

In this study, we analyzed differentially expressed miRNA for patients with high TMB and low TMB levels, and identified miRNAs that distinguish TMB-H from TMB-L tumor tissue samples. To the best of our knowledge, our study represents the first establishment of a four-miRNA-based signature classifier based on a training set consisting of 311 samples. The accuracy of the signature was validated in an independent test set consisting of 204 samples. ROC curves analysis revealed that, in the training set, the accuracy of the four-miRNA-based signature was 0.963; it was 0.902 in the test set; in the total set it was 0.946; this suggests the robustness of the classifier. Furthermore, the classifier had a high NPV, meaning that it has stronger recognition ability for low TMB. More importantly, MSI status weakly positively correlated with TMB levels, while the four-miRNA-based classifier tended to moderately positively correlated with TMB levels, which highlighted the potential advantage of using miRNA signature over MSI in predicting TMB.

We enrolled 27 patients with CRC and performed NGS and RT-PCR using FFPE tumor samples to validate the results. Consistent with previous results (24), CRC patients with MSI-H had significantly higher TMB than those with MSS. Importantly, the expression patterns of miRNAs were associated with TMB status. Upregulation of hsa-miR-625-3p, hsa-miR-552-5p, hsa-miR-592, and hsa-miR-224-5p were found in CRC samples as opposed to non-cancerous controls. Notably, relative expression levels of hsa-miR-625-3p and hsa-miR-552-5p in patients with TMB-H CRC were significantly higher than those with TMB-L CRC. Conversely, hsa-miR-592 and hsa-miR-224-5p expression levels were remarkably higher in patients with TMB-L CRC than in TMB-H CRC patients. Taken together, these findings suggest that the four-miRNA expression pattern could be applicable as potent predictor of TMB level in patients with CRC.

By inactivating mitogen activated protein kinase (MAPK) kinase MAP2K6, overexpression of miR-625-3p in CRC cell induced resistance to oxaliplatin-based therapy (33). Elevated expression levels of miR-552 were found in CRC tumor tissues (34). High expression levels of miR-552 promoted tumor growth in CRC, as demonstrated in proliferation and migration assays of CRC cells *in vitro* (35). The biological roles of miR-592 in CRC tumorigenesis have not been elucidated to date. We uncovered considerable upregulation of miR-592 in CRC tissues, compared to that in matched adjacent non-tumor tissues, suggesting miR-592 as a candidate potential oncogene in the pathogenesis of CRC. Another group identified miR-592 was a tumor suppressor in CRC, with significantly downregulated expression of miR-592 in the CRC tissue, in contrast to our findings (36). Nevertheless, several lines of evidence have demonstrated miR-592 may participate in CRC tumorigenesis (37, 38) and metastasis (39, 40). Furthermore, a study identified a potential role for miR-224 in CRC progression (41). Finally, high expression levels miR-224 have been associated with worse survival of patients in CRC (42).

In the current study, the results of enrichment analysis that evaluated the potential functions and pathways of the miRNA signature classifier were consistent with those of previous studies. The four-miRNA-based classifier correlated with TMB levels, in related to the weak correlation between MSI status and TMB levels. The result suggests that our methodology to evaluate TMB potentially facilitates the development of precision immunotherapy.

The strengths of this study include its design and its broad application prospects clinically. The robustness of four-miRNA-based signature classifier was validated using NGS and RT-PCR, adding strength to the findings. We also found that ROC analysis of four-miRNA expression value obtained from RT-qPCR exhibited similar AUCs from the testing and the training sets. The most widely investigated predictive biomarkers for TMB are MSI, and the four-miRNA classifier constructed by the LASSO method had a better performance than that of MSI. NGS is the gold standard for the detection of TMB; however, it is not a feasible or cost-effective method. Our classifier has clinical applicability for predicting TMB using conventional biopsies from CRC patients, which could be developed using traditional RT-qPCR. Moreover, as our classifier constructed by a small number of markers with high precise accuracy, further studies could verify these markers in clinical samples at relatively low cost. The application of the classifier has the potential to reduce associated health-care costs and may facilitate the selection of appropriate clinical management strategies. In the future, CRC patients would potentially select anti-PD-1 monotherapy for first-line treatment rather than combination chemotherapy according the stratification of the classifier; future confirmatory studies are required to validate this result.

Our study had several limitations that should be considered. First, our data were obtained from TCGA database; the epidemiology and distribution of clinical characteristics might be different in other areas. Multicenter and prospective studies with larger cohorts and other populations are needed to validate our results. Moreover, the biological mechanisms by which the four miRNAs are involved remain unknown; further insight into their functions might suggest novel treatment strategies. Finally, on account of the sample size, the TMB levels in the validation cohort were more positively correlated with MSI status than the four-miRNA-based classifier. Larger validation samples were needed to draw firmer conclusions.

To our knowledge, this is the first miRNA-based signature classifier validated by high-quality clinical data to accurately predict TMB level in patients with CRC. The findings demonstrated the discovery, assessment, and validation of a new classifier and emphasized that TMB is a core mechanism of sensitivity to checkpoint blockade in MSI-H CRC.

## Data Availability Statement

The datasets generated during and/or analysed during the current study are available from the corresponding author on reasonable request. All data generated or analysed during this study are included in this published article (and its supplementary information files).

## Ethics Statement

The study was approved by the Institutional Ethics Review Committee of Affiliated Tumor Hospital of Guangxi Medical University and First Affiliated Hospital of Guangxi Medical University. Prior to sample collection, each patient provided written informed consent.

## Author Contributions

WT and XP conceived the study. TL and JL carried out the experiments. HL and JL performed searches and analysis of the data. JH, HL, and YZ were involved in drafting the manuscript. All authors contributed to the article and approved the submitted version.

## Funding

This work was supported by the 2019 Guangxi University High-Level Innovation Team and the Project of Outstanding Scholars Program, and Guangxi Science and Technology Project (AD19245197).

## Conflict of Interest

The authors declare that the research was conducted in the absence of any commercial or financial relationships that could be construed as a potential conflict of interest.
