# Supplementary material for: MicroRNAs Expression Patterns Predict Tumor Mutational Burden in Colorectal Cancer
Source: Front Oncol. 2021 Feb 9;10:550986. doi: 10.3389/fonc.2020.550986 (PMC7900489; doi:10.3389/fonc.2020.550986)
Supplement: Supplementary file 1 [file Table_1.docx]

| Supplementary Table S1 Primers Used in the Quantitative Real-Time PCR | | | |
| --- | --- | --- | --- |
| Name | miRBase 21 ID | Direction | Sequence |
| hsa-miR-592 | MIMAT0003260 | F | CGCTTGTGTCAATATGCGATGATGT |
| hsa-miR-625-3p | MIMAT0004808 | F | CGGACTATAGAACTTTCCCCCTCA |
| hsa-miR-552-5p | MIMAT0026615 | F | CGGTTTAACCTTTTGCCTGTTGG |
| hsa-miR-224-5p | MIMAT0000281 | F | GTCAAGTCACTAGTGGTTCCGTTTAG |
| U6 |  | F | GGAACGATACAGAGAAGATTAGC |
