# Supplementary material for: MicroRNAs Expression Patterns Predict Tumor Mutational Burden in Colorectal Cancer
Source: Front Oncol. 2021 Feb 9;10:550986. doi: 10.3389/fonc.2020.550986 (PMC7900489; doi:10.3389/fonc.2020.550986)
Supplement: Supplementary file 2 [file Table_2.docx]

Supplementary Table S2 Performance of 4-miRNA-based classifier of TMB in CRC.

| Cohort | Se | Sp | PPV | NPV | Accuracy | AUC |
| --- | --- | --- | --- | --- | --- | --- |
| Training set | 0.967 | 0.901 | 0.907 | 0.965 | 0.907 | 96.33% |
| Test set | 0.857 | 0.881 | 0.878 | 0.86 | 0.878 | 90.24% |
| Total set | 0.938 | 0.881 | 0.887 | 0.934 | 0.890 | 94.60% |
